# Efficacy of Acupuncture for Mild to Moderate Depression in Older People: Protocol for a Randomized Controlled Trial

**DOI:** 10.2196/79327

**Published:** 2026-01-30

**Authors:** Qingnan Fu, Kaihui Xiao, Jie Zhang, Yang Li, Yuxian Wang, Molin Jiang, Zengqi Man, Jing Yang, Wei Lu

**Affiliations:** 1 Department of Psychosomatic Medicine, Beijing Traditional Chinese Medicine Hospital Affiliated to Capital Medical University, 23 Back Street, Art Museum, Dongcheng District, Beijing, China, 86 13801202917; 2 Neurology Ward 1, Dongzhimen Hospital, Beijing University of Chinese Medicine, Beijing, China; 3 Acupuncture and Massage College, Capital Medical University, Beijing, China

**Keywords:** depression, mild to moderate depression in older people, acupuncture, randomized controlled trial, protocol

## Abstract

**Background:**

Selective serotonin reuptake inhibitors are first-line antidepressants; however, only approximately 60% of patients can benefit from them. There is insufficient evidence for using acupuncture for symptom relief or for improving tolerance to selective serotonin reuptake inhibitors.

**Objective:**

This randomized controlled trial aims to assess the effects of acupuncture combined with citalopram hydrobromide on mild to moderate depression in older people.

**Methods:**

This study is a 2-arm, parallel, randomized controlled trial. A total of 132 participants aged 60 to 80 years diagnosed with major depressive disorder were divided into an acupuncture and medication group or a medication group. Participants in both groups take citalopram hydrobromide at a dose of up to 20 mg daily for 12 weeks. The acupuncture and medication group additionally receives 36 sessions of acupuncture treatment over 12 weeks. The primary outcome is the response rate of the 17-item Hamilton Depression Scale at the twelfth week. The secondary outcomes include changes in scores on the 17-item Hamilton Depression Scale and Mini-Mental State Examination at various time points. Adverse events will be recorded in detail.

**Results:**

The study commenced on June 30, 2023, and as of October 17, 2024, a total of 132 participants had been enrolled. Data collection has been completed. Currently, data analysis is in progress, with preliminary findings anticipated to be available by October 2025. The findings of this study are expected to be submitted for publication in 2026.

**Conclusions:**

This pilot study is expected to provide critical insights into the feasibility of integrating acupuncture with standard medication for managing mild to moderate depression in older people. By generating preliminary evidence on its potential benefits, the study aims to inform the design and sample size estimation of future multicenter trials, potentially advancing nonpharmacological treatment options for depression.

## Introduction

Depression is a common mental disorder caused by an interaction of social, psychological, and biological factors that can occur in anyone [1]. The clinical symptoms include anhedonia, fatigue, and negative emotions [2,3], as well as increased vulnerability to physical illnesses such as stroke, pain, and cancer [4,5], which may contribute to poor responses to pharmacological treatments. The World Health Organization predicted that it would become the greatest cause of global disease burden by 2030 [6]. Depression is a major issue for older adults, and its prevalence is rising [7]. Old age is the time of life when emotional fragility is accentuated. In addition to neurobiological changes in the brain, aging inevitably entails significant losses over time, not only in terms of individuals’ emotions but also in terms of their physical condition and social status. Depression is the most common psychological disorder among people older than 65 years and affects approximately 15% of this age group [8].

Currently, drug therapy and psychotherapy are the main forms of treatment for depression, with various complementary therapies serving as an adjunct. The standard drug treatments (mainly selective serotonin reuptake inhibitors [SSRIs]), such as paroxetine, sertraline, and citalopram, are not entirely satisfactory. The response rate to SSRIs, defined as at least a 50% improvement from baseline, is only approximately 60%, with documented side effects such as nausea, weight gain, and reduced sexual function, which limit patient compliance with the treatment [9,10]. As a special group, older adults require safer support options due to their low tolerance to medications, poor compliance with psychotherapy, and higher rates of chronic physical illnesses [11]. Therefore, new strategies to enhance the efficacy of antidepressants in the early stage of treatment are urgently needed.

Acupuncture treatment for depression has been provided alone or together with antidepressants in China and worldwide for years. Growing evidence indicates that acupuncture combined with drug treatment is more effective than drug treatment alone, is safe and well tolerated, and has an early onset of action [12]. However, systematic reviews have shown the potential benefits of acupuncture as an add-on therapy, but they have failed to obtain high-certainty evidence. The main limits of past studies have been a lack of large sample sizes, a high risk of bias, and inconsistent results [13-15].

This randomized controlled trial (RCT) is designed to evaluate the difference in efficacy between citalopram hydrobromide alone versus citalopram hydrobromide together with acupuncture for mild to moderate depression in older people.

## Methods

### Study Design

**Figure 1.**
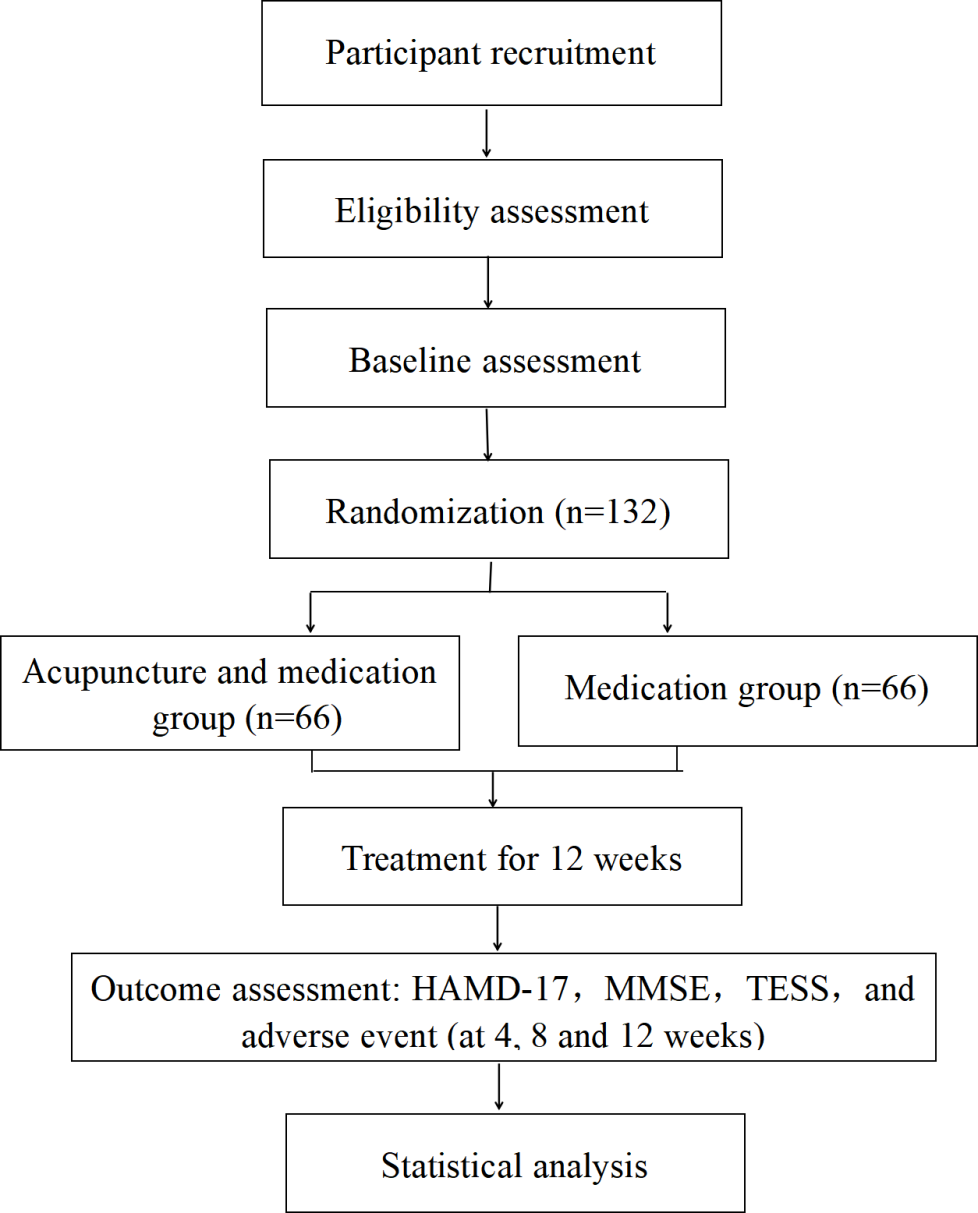
Flowchart of the trial design. HAMD-17: 17-item Hamilton Depression Scale; MMSE: Mini-Mental State Examination; TESS: Treatment Emergent Symptom Scale.

This prospective RCT was performed at the Department of Psychosomatic Medicine in Beijing Traditional Chinese Medicine Hospital Affiliated to Capital Medical University between January 2022 and December 2024. The protocol was developed in accordance with the SPIRIT (Standard Protocol Items: Recommendations for Interventional Trials) 2013 checklist [16] and the STRICTA (Standards for Reporting Interventions in Clinical Trials of Acupuncture) guidelines [17]. The trial was registered with the Chinese Clinical Trial Registry (ChiCTR2300072740) in June 2023, prior to the enrollment of the first participant (on June 30, 2023). A flowchart of the trial design is shown in Figure 1, and the study timeline is presented in Table 1.

**Table 1.** Schedule of recruitment, interventions, and outcome measurements.

	Treatment period			
	Baseline	Week 4	Week 8	Week 12
Enrollment				
Screening	✓			
Signing of informed consent	✓			
Randomization	✓			
Intervention				
Acupuncture and medication group		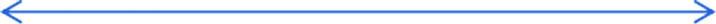		
Medication group		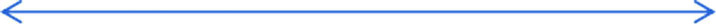		
Outcome measurements				
Primary outcome				
HAMD-17^a^	✓	✓	✓	✓
Secondary outcomes				
MMSE^b^	✓	✓	✓	✓
TESS^c^	✓	✓	✓	✓
Routine blood test, routine urine test, routine stool test, liver function test, renal function test, and an ECG^d^	✓			✓
Adverse events		✓	✓	✓

a HAMD17: 17-item Hamilton Depression Scale.

b MMSE: Mini-Mental State Examination.

c TESS: Treatment Emergent Symptom Scale.

d ECG: electrocardiogram.

### Study Setting and Recruitment

A total of 132 participants were recruited through posters and hospital websites, and they were allocated to 2 parallel treatment groups using a 1:1 allocation ratio. Participants were assessed at the following time points: baseline, the middle of the treatment (4 and 8 weeks after treatment starts), and the end of the treatment (12 weeks after treatment starts).

### Inclusion Criteria

The inclusion criteria were as follows: (1) participants who met the criteria for major depressive disorder according to the *International Classification of Diseases, Tenth Revision*; (2) men or women aged 60 to 80 years; (3) a 17-item Hamilton Depression Rating Scale (HAMD-17) score of 7 to 24; (4) participants who were consistently not using antidepressants or had already stopped taking antidepressants for more than 4 weeks; (5) participants who did not receive acupuncture treatment in the last 4 weeks and did not participate in any other ongoing research; and (6) participants who provided informed consent.

### Exclusion Criteria

Participants who reported any of the following conditions were excluded: (1) fundamental communication problems, (2) high risk of suicide or self-injurious behavior, (3) dementia, (4) a history of bipolar disorder, (5) a history of schizophrenia or psychotic symptoms, (6) current or lifetime alcohol abuse, and (7) severe cardiovascular diseases or poor liver or kidney function.

### Randomization and Blinding

Eligible participants were randomly assigned to either the acupuncture and medication group or the medication group in a 1:1 ratio. To ensure equal distribution between treatment groups, the random block size was set to a fixed size of 6. An independent statistician generated a block randomization schedule using SAS (version 9.4; SAS Institute Inc). The randomization scheme, protected by strict viewing permission, is kept as a blind code by a member of staff who is not involved in this study. Baseline assessors were responsible for randomization. They obtained a random number and group assignment over the telephone. Patients and acupuncturists were not blinded. Outcome assessors and statisticians who perform the statistical analyses are blinded to group assignment. Participants’ allocated intervention will not be revealed until the statistical analysis is completed.

### Interventions

#### Acupuncture and Medication Group

The selection of acupoints was based on our previous study on major depression, clinical experience, and expert opinion. Treatment was performed by licensed acupuncturists who had at least 5 years of experience in acupuncture. All acupuncturists were trained on how to locate acupoints, puncture, and manipulate needles before the trial. Sterile disposable acupuncture needles (length: 25‐40 mm, diameter: 0.25 mm; Hua Tuo) will be used. Acupuncture treatments consisted of 36 sessions with a 30-minute duration over 12 weeks (3 sessions per week, ideally every other day). Acupuncture was discontinued if patients experienced any adverse events (AEs).

**Figure 2.**
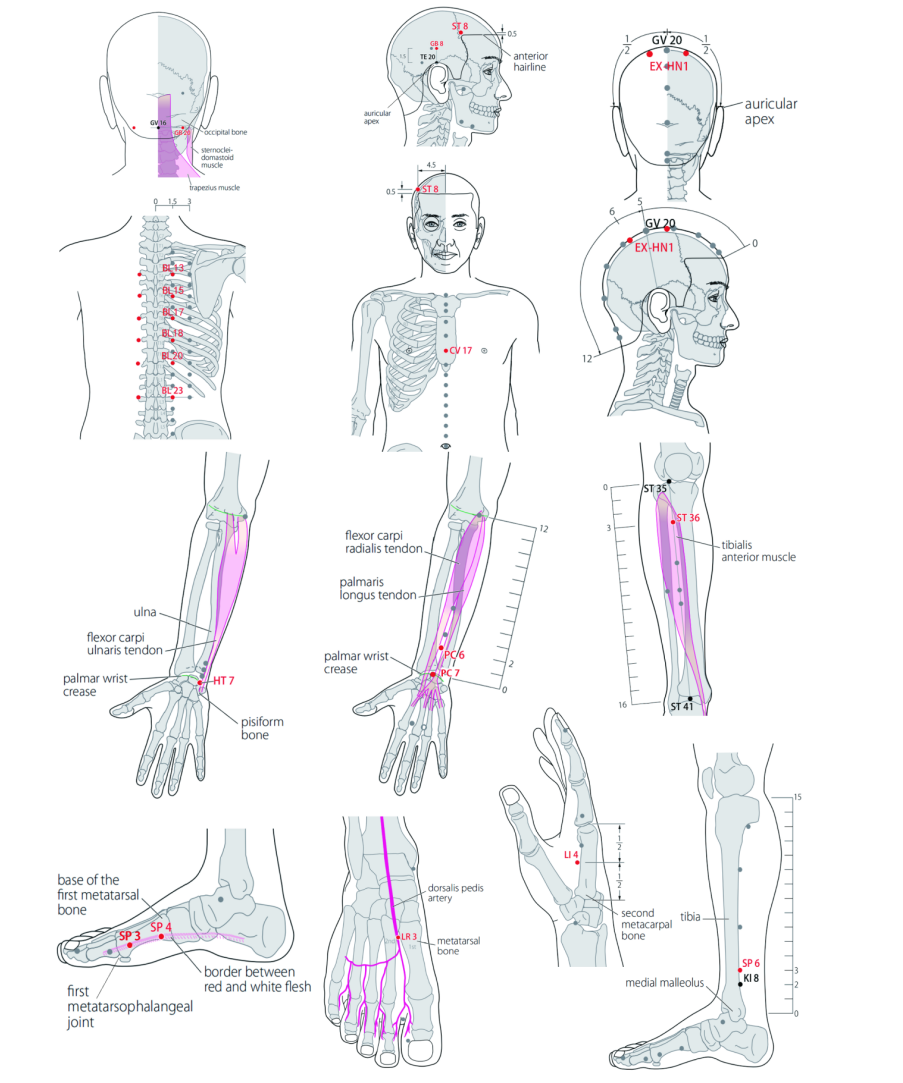
Acupoints selected for use in the study were localized according to the World Health Organization Standard Acupuncture Locations.

Participants allocated to the acupuncture and medication group received treatment using needles inserted at the prespecified acupuncture points. The protocol, including obligatory and additional acupoints, was developed from the clinical experience of acupuncture experts. The obligatory acupoints included bilateral *feishu* (BL13), *xinshu* (BL15), *ganshu* (BL18), *pishu* (BL23), *shenshu* (BL35), and *geshu* (BL17). According to different symptoms, additional acupoints could be chosen individually: *fengchi* (G20) and *taichong* (LR3) for dizziness, *shenmen* (TF4) and *sishencong* (Ex-HN1) for sleep disorders, *touwei* (SRT8) and *shuaigu* (GB8) for headache, *zusanli* (ST36) and *sanyinjiao* (SP6) for lassitude, *hegu* (LI4) and *daling* (PC7) for anxiety, *taibai* (SP3) and *gongsun* (SP4) for inappetence, and *neiguan* (PC6) and *danzhong* (RN17) for heart palpitations and chest tightness. All acupoints were localized according to the World Health Organization Standard Acupuncture Locations [18] (Table 2 and Figure 2). Manipulations of twirling, lifting, and thrusting were performed on all needles to reach *deqi* (a composite sensation including soreness, numbness, distention, and heaviness), which is believed to be an essential component of acupuncture efficacy.

**Table 2.** Acupoints selected for use in the study.

Acupoints	Location
Ganshu (BL18)	In the upper back region, at the same level as the inferior border of the spinous process of the ninth thoracic vertebra, 1.5 B-*cun* lateral to the posterior median line.
Shenshu (BL23)	In the lumbar region, at the same level as the inferior border of the spinous process of the second lumbar vertebra, 1.5 B-*cun* lateral to the posterior median line.
Pishu (BL20)	In the upper back region, at the same level as the inferior border of the spinous process of the 11th thoracic vertebra, 1.5 B-*cun* lateral to the posterior median line.
Xinshu (BL15)	In the upper back region, at the same level as the inferior border of the spinous process of the fifth thoracic vertebra, 1.5 B-*cun* lateral to the posterior median line.
Feishu (BL13)	In the upper back region, at the same level as the inferior border of the spinous process of the third thoracic vertebra, 1.5 B-*cun* lateral to the posterior median line.
Geshu (BL17)	In the upper back region, at the same level as the inferior border of the spinous process of the seventh thoracic vertebra, 1.5 B-*cun* lateral to the posterior median line.
Fengchi (G20)	In the anterior region of the neck, inferior to the occipital bone, in the depression between the origins of the sternocleidomastoid and the trapezius muscles.
Taichong (LR3)	On the dorsum of the foot, between the first and second metatarsal bones, in the depression distal to the junction of the bases of the 2 bones, over the dorsalis pedis artery.
Shenmen (TF4)	On the anteromedial aspect of the wrist, radial to the flexor carpi ulnaris tendon, on the palmar wrist crease.
Sishencong (Ex-HN1)	On the parietal region, 1 *cun* anterior, posterior, and lateral to *baihui*; 4 acupoints.
Touwei (SRT8)	On the head, 0.5 B-*cun* directly superior to the anterior hairline at the corner of the forehead, 4.5 B-*cun* lateral to the anterior median line.
Shuaigu (GB8)	On the head, directly superior to the auricular apex, 1.5 B-*cun* superior to the temporal hairline.
Zusanli (ST36)	On the anterior aspect of the leg, on the line connecting ST35 with ST41, 3 B-*cun* inferior to ST35.
Sanyinjiao (SP6)	On the tibial aspect of the leg, posterior to the medial border of the tibia, 3 B-*cun* superior to the prominence of the medial malleolus.
Hegu (LI4)	On the dorsum of the hand, radial to the midpoint of the second metacarpal bone.
Daling (PC7)	On the anterior aspect of the wrist, between the tendons of palmaris longus and the flexor carpi radialis, on the palmar wrist crease.
Taibai (SP3)	On the medial aspect of the foot, in the depression proximal to the first metatarsophalangeal joint, at the border between the red and white flesh.
Gongsun (SP4)	On the medial aspect of the foot, anteroinferior to the base of the first metatarsal bone, at the border between the red and white flesh.
Neiguan (PC6)	On the anterior aspect of the forearm, between the tendons of the palmaris longus and the flexor carpi radialis, 2 B-*cun* proximal to the palmar wrist crease.
Danzhong (RN17)	In the anterior thoracic region, at the same level as the fourth intercostal space, on the anterior median line.

Participants took citalopram hydrobromide up to 20 mg daily. Patients’ usual treating clinicians adjusted doses based on their routine practices.

#### Medication Group

Participants in this group took the same medication at the same dose and schedule as the acupuncture and medication group. Additional treatment for depression was not provided.

### Outcome Measurements

Follow-up examinations were performed at baseline and at 4, 8, and 12 weeks after treatment initiation in the 2 groups.

### Primary Outcome

The primary outcome measurement of this study is the clinical response rate according to the total HAMD-17 score at the end of the study. Response is defined as a 50% or greater decrease from baseline in the total HAMD-17 score at the end of the study (week 12). The HAMD-17 is a multiple-item questionnaire used to assess the severity of depression and as a guide to evaluate recovery. The range of the HAMD-17 score is 0 to 52, with 0 to 6 indicating normal depression, 7 to 17 indicating mild depression, 18 to 24 indicating moderate depression, and 25 or more indicating severe depression [19].

### Secondary Outcomes

#### Hamilton Depression Scale

Changes in the total HAMD-17 score from baseline are assessed.

#### Mini-Mental State Examination

The Mini-Mental State Examination (MMSE) is used to screen for possible cognitive impairment. The MMSE contains 30 items that assess a range of cognitive functions, such as orientation, short-term memory, language, comprehension, attention, and calculation. Total scores on the MMSE range from 0 to 30. Participants with scores of 23 or below are considered likely to have a cognitive impairment [20].

#### Treatment Emergent Symptom Scale

The Treatment Emergent Symptom Scale is commonly used to measure the presence and intensity of psychotropic medication side effects [21,22]. Both intensity (from 0 to 4, with a higher score correlating with a worse condition) and intervention (from 0 to 6) are documented in the scale.

### Safety Assessment

A routine blood test, routine urine test, routine stool test, liver function test, renal function test, and an electrocardiogram were administered for safety outcomes. These biological indicators were monitored during the screening period and after 12 weeks of treatment.

The most common AEs related to acupuncture treatment include localized hematoma, localized infection, broken needles, fainting, bleeding, nausea, dizziness, vomiting, palpitations, and severe pain. Detailed information on AEs and serious AEs (SAEs), including the name, onset and end date, intensity, relationship with acupuncture, and outcome, is recorded. If the treatments cause SAEs, researchers will immediately report them to the medical ethics committee of Beijing Traditional Chinese Medicine Hospital and suspend the study.

### Sample Size Calculation

The sample size was calculated based on a noninferiority design using PASS (version 15.0; NCSS, LLC). The primary objective is to demonstrate that acupuncture combined with medication is not inferior to medication alone. We conservatively assumed a baseline rate of 30% for the control group (medication alone). This assumption aligns with large-scale clinical trials such as the Sequenced Treatment Alternatives to Relieve Depression (STAR*D) study [23], which reported a remission rate of approximately 30% to 33% for patients treated with citalopram. For the intervention group, based on our pilot data and clinical experience, we anticipated a response rate of 40%. It is important to note that while this anticipation was used for power calculation to ensure adequate sensitivity, the hypothesis testing remains focused on noninferiority. The noninferiority margin (Δ) was set at −15%. This margin was chosen based on the risk-benefit profile: given that acupuncture is a physical therapy with a superior safety profile compared to pharmacotherapy, a wider margin is clinically acceptable. With a 1-sided significance level (α) of .025 and 80% power, a sample size of 57 patients per group was required. Assuming a dropout rate of 15%, we aimed to recruit a total of 132 participants (66 per group).

### Statistical Analysis

The statistical analysis is performed by an independent statistician who is not aware of the group allocation. SAS (version 9.4) is used for data analysis. The level of significance is set at α <.05 with a 2-sided test. Continuous data are represented as the mean (SD) or median (IQR), whereas categorical data are represented as percentages.

All efficacy analyses are performed using the intent-to-treat approach. For the intent-to-treat analysis, the population consists of all patients who have been randomized. Missing data are imputed using the multiple imputation method. Continuous variables are compared using the Student *t* test or Wilcoxon rank sum test, as appropriate. Categorical variables are compared using the Fisher exact test or the Wilcoxon rank sum test, as appropriate.

Our primary analysis for between-group differences uses a repeated measures ANOVA (or a mixed effects model) to account for the multiple time points (weeks 0, 4, 8, and 12), with “group” as the between-subject factor and “time” as the within-subject factor. This model appropriately handles the longitudinal nature of the data. We have also specified that the primary end point is the difference at week 12.

A sensitivity analysis is being conducted for the primary outcome using the per-protocol population, including only those patients who complete at least 80% of allocated treatments and have no major protocol violations (taking other drugs during the trial or not completing the case report form as required).

### Data Monitoring and Quality Control

All researchers, including acupuncturists, outcome assessors, and statisticians, receive training regarding data management. Monitors check case report forms once every week as well as the acupuncture operation during the treatment period. Dropouts and withdrawals, including the reasons, are documented in detail throughout the trial. Participants’ information is stored in locked file cabinets at the study sites with limited access; only investigators have the right to access the data.

### Data Availability Statement

All data will be preserved for at least 5 years after publication. The principal investigator will have access to the final trial dataset, and trial data will be made available to readers upon reasonable request by contacting the corresponding author. Information on patients will remain anonymous, including name, age, and telephone number.

### Patient and Public Involvement Statement

Patients were not involved in the design, conduct, or reporting of this trial. They will be evaluated for their ability to complete the study treatment based on their time and the degree of mobility difficulty. Specifically, we consulted patients before the trial regarding the burden of 36 sessions. On the basis of their feedback, we implemented the following measures: (1) flexible scheduling—avoiding early morning sessions and allowing makeup visits, (2) caregiver engagement—actively involving family members to support adherence and (3) outcome relevance—confirming that our chosen outcomes aligned with patients’ daily living priorities. A journal article manuscript will be prepared to present the results after the trial is completed, and a brief summary of the results in plain language will be provided to all participants. No attempt will be made to assess the burden of the intervention on the patients themselves.

### Ethical Considerations

The trial protocol (version 2.0; September 19, 2022) was planned in accordance with the Declaration of Helsinki and has been approved by the ethics committee of Beijing Traditional Chinese Medicine Hospital Affiliated to Capital Medical University (2022BL02-061-01). The trial was registered in June 2023 at the Chinese Clinical Trial Registry (ChiCTR2300072740). Before each participant enrolled in this study, the investigator provided a complete and comprehensive written introduction to the purpose, procedures, and potential risks of the study. All the participants were fully informed about this trial and given enough time to inquire about details and decide whether to participate at the first visit. Participants were asked to sign the informed consent form if they agreed to participate. Participants were informed of their right to withdraw from the study at any time. If a participant withdrew during the course of the trial, we reserved the right to retain the data obtained for statistical analysis. All data are anonymized. All participants were entitled to take free citalopram hydrobromide (up to 20 mg) daily for 12 weeks, and participants in the acupuncture and medication group were entitled to 36 free acupuncture treatments. No other compensation, such as for transportation or lost wages, was provided. Trial results will be published in peer-reviewed journals and will be disseminated to the media and the general public.

## Results

The study commenced on June 30, 2023, and as of October 17, 2024, a total of 132 participants had been enrolled. Data collection has been completed. Currently, data analysis is in progress, with preliminary findings anticipated to be available by October 2025. The findings of this study are expected to be submitted for publication in 2026.

## Discussion

### Anticipated Findings

This is an RCT designed to investigate the efficacy of acupuncture for mild to moderate depression in older people over a 12-week period. The trial is designed to meet the methodological demands of adequate power and allocation concealment, following the Good Clinical Practice guideline [24]. This study is defined as a “pilot” or “preliminary” trial because it is the first step in our research program to evaluate the specific integration of acupuncture with citalopram for the older population. Its main purposes are to generate effect size estimates for sample size calculation of future multicenter trials and to test the feasibility of the protocol (including recruitment and retention) in a single center. The results of this study may provide further evidence for the effectiveness of acupuncture in promoting depressive emotion and improving cognitive function in older people with mild to moderate depression.

Acupuncture has been used to treat mental disorders since antiquity in China. Results from animal experiments suggest a multitarget antidepressant effect of acupuncture, which may be related to amino acid metabolism and inflammatory pathways, especially the toll-like receptor signaling pathway, tumor necrosis factor signaling pathway, and nuclear factor κ light chain enhancer of activated B cells signaling pathway [25]. The literature has emphasized that acupuncture combined with antidepressant pharmacological treatment not only enhances the improvement of primary and secondary depressive symptoms but also reduces the side effects of medical treatment, which is the main cause of high dropout rates for drug treatment [26]. Emotional and cognitive disorders are common psychological features underlying the depressive symptoms of depression. It has been found that those with depression have difficulty shifting attention from negative information, which leads to persistent negative emotional rumination. Depression in older adults is frequently comorbid with cognitive decline (“pseudodementia”), and improvement in depressive symptoms often correlates with cognitive recovery. The existing studies on depression are mainly based on responses to clinical scales to assess depressive symptoms but have not focused on the effects of acupuncture on cognition in those with depression. However, the mechanisms of how acupuncture regulates attentional bias in those with depression are still not clear. Therefore, this trial is designed to explore the clinical efficacy and safety of acupuncture therapy for mild to moderate depression in older people. Moreover, we will use indices reflective of cognitive function, such as the MMSE, as secondary outcomes, with the expectation that they will provide reliable clinical evidence for acupuncture treatment that helps improve depression, which is a key concern for the older population.

However, there are several limitations that need to be acknowledged. First, the single-center experimental design will result in a single sample, with limited representativeness, and possible experimental bias. More influencing factors should be considered, and the result should be further verified and explored in a large sample population. Second, blinding of acupuncturists is not possible due to the unique nature of acupuncture procedures. To reduce the impact, we provided detailed training to the acupuncturists, including standardized communication with patients. Third, the decision not to use a sham control is supported by public concerns on the methodology of sham or placebo acupuncture design, as well as the substantial risk of failure in blinding of participants, who are usually very experienced with acupuncture treatment in Chinese medical culture and settings. Finally, without a follow-up period, it will be impossible to assess how long the therapeutic effect of acupuncture lasts. However, as a preliminary trial, our primary goal is to establish immediate efficacy and feasibility at the end of the 12-week treatment. This phased approach aligns with standard research frameworks for complex interventions [27]. Previous pilot studies [28] have similarly focused on end-of-treatment outcomes to validate protocols before extending to longitudinal follow-up. Future confirmatory trials will include a 3-month follow-up.

### Conclusions

This pilot study is expected to provide critical insights into the feasibility of integrating acupuncture with standard medication for managing mild to moderate depression in older people. By generating preliminary evidence on its potential benefits, the study aims to inform the design and sample size estimation of future multicenter trials, potentially advancing nonpharmacological treatment options for depression.

## Abbreviations

AE: adverse event
HAMD-17: 17-item Hamilton Depression Scale
MMSE: Mini-Mental State Examination
RCT: randomized controlled trial
SAE: serious adverse event
SPIRIT: Standard Protocol Items: Recommendations for Interventional Trials
SSRI: selective serotonin reuptake inhibitor
STRICTA: Standards for Reporting Interventions in Clinical Trials of Acupuncture
